# Marine Waste—Sources, Fate, Risks, Challenges and Research Needs

**DOI:** 10.3390/ijerph18020433

**Published:** 2021-01-07

**Authors:** Jolanta Dąbrowska, Marcin Sobota, Małgorzata Świąder, Paweł Borowski, Andrzej Moryl, Radosław Stodolak, Ewa Kucharczak, Zofia Zięba, Jan K. Kazak

**Affiliations:** 1 Institute of Building Engineering, Wrocław University of Environmental and Life Sciences, 50-363 Wrocław, Poland; zofia.zieba@upwr.edu.pl; 2 Institute of Landscape Architecture, Wrocław University of Environmental and Life Sciences, 50-357 Wrocław, Poland; marcin.sobota@upwr.edu.pl; 3 Institute of Spatial Management, Wrocław University of Environmental and Life Sciences, 50-357 Wrocław, Poland; malgorzata.swiader@upwr.edu.pl (M.Ś.); jan.kazak@upwr.edu.pl (J.K.K.); 4 Faculty of Marine Engineering, Maritime University of Szczecin, 71-650 Szczecin, Poland; p.borowski@am.szczecin.pl; 5 Institute of Environmental Engineering, Wrocław University of Environmental and Life Sciences, 50-363 Wrocław, Poland; andrzej.moryl@upwr.edu.pl (A.M.); radoslaw.stodolak@upwr.edu.pl (R.S.); 6 Department of Pharmacology and Toxicology, Wrocław University of Environmental and Life Sciences, 50-375 Wrocław, Poland; ewa.kucharczak@upwr.edu.pl

**Keywords:** microplastics, nanoplastics, marine transport, ship recycling, radioactive waste, sustainable waste management, chemical weapon, SARS-CoV-2, MARPOL, marine ecosystems

## Abstract

The article presents a comprehensive and cross-cutting review of key marine waste issues, taking into account: sources, fate, risks, transport pathways, threats, legislation, current challenges, and knowledge gaps. The growing amount of both human-created waste in seas and oceans and waste reaching marine ecosystems from land is one of today’s challenges for the global economy and the European Union. It is predicted that if no decisive steps are taken to limit the amount of this type of waste, there may be more plastic waste than fish in the oceans after 2050. The influence of microplastics and nanoplastics on living organisms remains undiagnosed. Within the international and EU law, solutions are being developed to properly manage waste on board ships and to reduce the impact of processes related to the recycling of the vessels on the environment. Currently, over 80% of ships are dismantled in the countries of South Asia, in conditions that threaten the environment and the safety of workers. After World War 2, large quantities of chemical weapons were deposited in the seas. Steel containers with dangerous substances residing in the sea for over 70 years have begun leaking, thus polluting water. For many years, radioactive waste had also been dumped into marine ecosystems, although since 1993 there has been a total ban on such disposal of radionuclides. The impact of the COVID-19 pandemic on marine waste generation has also been presented as a significant factor influencing marine waste generation and management.

## 1. Introduction

Marine ecosystems are highly exposed to contamination from human activities. Recently, a growing number of emerging pollutants (i.e., nanoplastics and microplastics) have been reaching the pristine environments of the Arctic or the Antarctic [1,2,3,4,5]. For years the oceans have been targeted as a dumping site of industrial waste, chemicals (including chemical weapons), wastewaters, garbage, and other waste from terrestrial sources. Dumping of some substances, i.e., radionuclides, has been banned in recent years; nevertheless, they still remain in the environment [6,7,8].

The development of modern remote sensing techniques, easier access to spatial data, environmental quality data, and progress in analytical chemistry are all contributing to the broadening of knowledge on marine waste. However, there are still serious shortages of knowledge and information related to the magnitude, location, and temporal variability of the waste sources and pathways, fate of waste, biological and chemical interactions in the environment [9,10,11,12]. Problems of a legal nature are common in the management of waste that poses a threat to the seas—the lack of appropriate regulations, the vagueness of or non-compliance with existing laws, the lack of or inadequate policies and strategies [13,14,15,16].

While chemical weapons and radioactive substances no longer enter the seas and oceans in large quantities, plastics waste still poses a major threat to marine ecosystems.

With rapid population growth, and raising living standards and consumption levels, annual plastic waste generation is increasing year after year. World production of plastic materials has been growing for many years. Due to its beneficial properties and low production cost, plastic has been displacing traditional, natural materials. Unfortunately, as it becomes waste, it constitutes a great threat to the environment. Of the 350 million tonnes of annual world plastic, over 90% is not recycled, and it is estimated that 10% ends up in the seas and oceans, accounting for 85% of marine waste [9,17,18,19,20,21].

The problem of marine debris (consisting mainly of plastic) was noticed in the 1990s [22]. The results of research conducted by a number of authors indicate that 80% of marine debris comes from land-based sources, and 20% are ship-sourced. More than 636,000 tonnes of waste per year are brought into the sea from ships, and the greatest threat to the marine environment is posed by cruise vessels [23,24].

As part of the prevention of marine plastic pollution, new legal frameworks and strategies are being developed. Such actions are undertaken by the European Union, the United Nations, and the Group of Twenty (G20). The G20, which includes the leading plastic manufacturers: China, US, and Germany, is responsible for two-thirds of global plastic waste, created “G20 Action Plan on Marine Litter” [25,26]. The most important aspect here is plastic waste management through a circular economy approach. Environmentally Sound Waste Management (ESWM) must ensure the lack of possibility of plastic escape from the economic system (at the stage of: product design, manufacturing and service delivery, distribution and use, as well as end-of-life management). Government policy should include, but not be limited to, creating regulations prohibiting the use of non-recyclable plastic materials and supporting bio-based alternatives, limiting the misuse of plastic waste in the formal and informal sector, employing extended producer responsibility, and supporting marine protection and conservation plans. It is also crucial to create instruments and programs for knowledge and innovation development (i.e., R&D projects) and to co-create collective policy initiatives and actions by all stakeholders, including producers, consumers, non-governmental organizations, representatives of the informal sector, policymakers, retailers, trade associations, recyclers, municipalities, and national authorities [25,26,27].

Research related to water protection is currently focused on urgent topics: eutrophication, population growth and climate change, plastic waste, creating new legal solutions, and decision support systems for effective water protection. The mutual interactions between these problems, which are quite rarely described in a broad sense and in relation to marine waters, are important, as most studies concern freshwater ecosystems or connect a few of the abovementioned issues [5,14,28,29,30,31,32].

In 2019, the SARS-CoV-2 virus was identified in China, and the year 2020 witnesses a rapid growth of the COVID-19 pandemic which has had a significant impact on the economy and thus on the pollution of the seas and oceans. According to many authors and sources, the COVID-19 pandemic has both positive and negative influences on the environment. Frozen anthropogenic activity has reduced emissions of some pollutants [33,34], but also increased the amount of waste from Personal Protective Equipment (PPE) [35,36].

Research conducted by Patrício Silva et al. [37] indicates that due to the COVID-19 pandemic we are dealing with increased plastic pollution. At the same time, the authors of this article emphasize that the number of studies in environmental sciences related to the COVID-19 pandemic is significantly smaller than in other fields, they constitute less than 3% of all studies. Moreover, only about 20% articles in environmental sciences concern the problem of waste. Compared to the area of medicine and health, there is a deficit of publications related to the environmental impact of the COVID-19 pandemic.

The aim of the paper is to widely present the problem of marine waste, taking into account the most important sources of threats for the seas and oceans, as well as the legislation, current issues and hotspots, challenges, risks, and knowledge gaps.

The article has been divided into five thematic parts reflecting current problems for the studied issue: 1. Plastic debris in the marine environment, 2. Ship industry and marine pollution (ship waste and ships as waste), 3. Chemical weapon munitions dumped at sea, 4. Radioactive waste dumping at sea, 5. COVID-19 pandemic impact on marine waste generation.

## 2. Methods and Data Selection

The review of the current state of knowledge considering all marine wastes’ aspects mentioned above was conducted mainly based on scientific databases as well as reports of international and non-governmental organizations (NGOs).

The following scientific literature databases were selected for review: Science Direct, Web of Science, Scopus, and Google Scholar. For topics 1, 3, and 4 search strings: “microplastic”, “nanoplastics”, “plastic debris”, “litter”, “waste”, “chemical weapon”, “munitions”, “radionuclides”, “radioactive waste”, and “nuclear waste” were used. The research resulted from this search was filtered to articles concerning marine ecosystems, thus narrowing the collection to those containing keywords: “marine”, “sea”, and “ocean”. For topic 2, the following keywords were used: “waste management on board ships”, “ship recycling”, “ship waste”. In the last stage, for issue 5, the articles containing keywords “COVID-19 pandemic” and “SARS-CoV-2” were extracted from the results of the previous searches. The search was conducted for all fields.

The same search scheme was used in the top search engine–Google Search. From the records, the reports of intergovernmental and international organizations that are the most important for the studied area were chosen, i.e., of International Maritime Organization (IMO) and Marine Environment Protection Committee (MEPC), The Baltic Marine Environment Protection Commission–also known as the Helsinki Commission (HELCOM), The European Maritime Safety Agency (EMSA), International Atomic Energy Agency (IAEA), European Centre for Disease Prevention and Control–an agency of the European Union (ECDP). Moreover, reports from NGOs which focus on marine waste in their activity and important press releases were taken into account.

The final stage was to analyze the current legal status of the five thematic parts, paying special attention to international and EU regulations.

For research resulting from the search, relevancy assessment and quality evaluation were carried out. Consequently, 122 articles, books, book chapters, reports, maps, and legal acts from 1973–2021 were selected.

## 3. Plastic Debris in the Marine Environment

**Figure 1:**
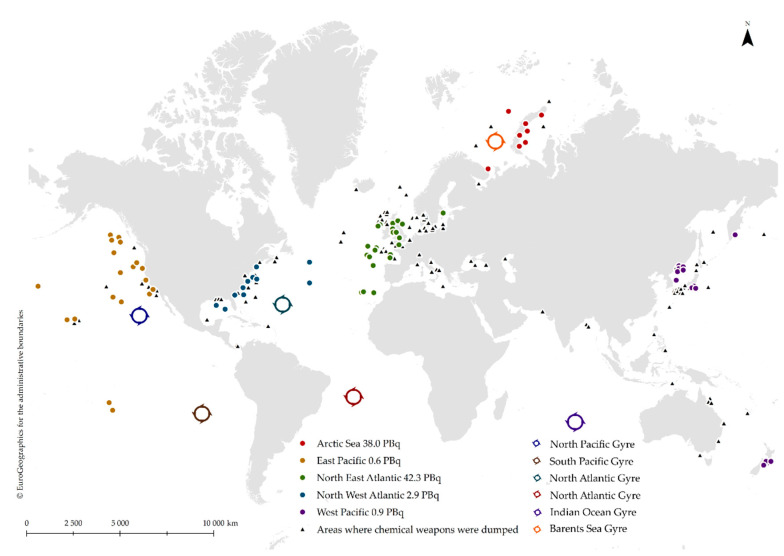
The location of ocean garbage patches, the global inventory of radioactive waste disposal at sea (total amounts in petabequerels per region) and chemical weapons dumped at sea. Data sources: [7,40,41,42], administrative boundaries in shapefile format were obtained from Eurostat’s geodata database [43].

Plastic waste enters the sea from numerous land and sea-based sources and has different sizes and transport mechanisms. Popular macroplastics include: plastic bags and bottles (from households, streets, coastal tourism, ships via direct or indirect entry—transported by wind, rivers, tides, and drift), cotton bud sticks (from households via sewage systems and rivers or tides), fishing nets and lines, injection gun cartridges and plastic sheetings to protect mussel cultures (from fishery and aquaculture via direct entry—transported by drift and tides) [38]. The Great Pacific Garbage Patch (GPGP) is the largest of the five offshore plastic accumulation zones in the world’s oceans (Figure 1). Research carried out by Lebreton et al. proved that GPGP consists of 79 (45–129) thousand tonnes of plastic, covering the area of 1.6 million km^2^. Over 75% of the GPGP mass was carried by debris larger than 5 cm and at least 46% was comprised of fishing nets. Microplastics accounted for 8% of the total mass but 94% of the estimated 1.8 (1.1–3.6) trillion pieces floating in the area [9]. There are currently about 150 million tonnes of plastics in the oceans. Possible scenarios indicate that without decisive action to protect marine ecosystems, one tonne of plastic will correspond to every 3 tonnes of fish by 2025, and by 2050, we can expect more plastic than fish (by weight). If no action is taken, the global amount of plastics that enter the oceans is projected to double by 2030 and quadruple by 2050 [25,39].

Microplastics are defined as plastic debris having particles with at least one dimension <5 mm, and particles <1 µm are known as nanoplastics [44,45,46]. Microplastics (MPs) and nanoplastics (NPs) have been recently detected in the atmosphere [47,48], freshwater and drinking water resources [28,49,50], wastewater and sewage sludge [51], soils and plants [52,53,54,55], marine ecosystems [17,45], in human and animal organisms [18,56,57], and even in the waters of the Arctic and the Antarctic [2,3,58].

Microplastics in the environment come from primary and secondary sources. Primary sources are industrial materials, personal care products (i.e., exfoliating facial cleansers), and cleaning products additives. MPs are also used in medicine as vectors for drugs. Primary microplastics include pellets, beads, nurdles, fibers, and powders, and as regards the material, they are usually polyethylene (PE), polypropylene (PP), and polystyrene (PS). Secondary microplastics are created from the breakdown of larger plastic debris, mainly as a result of weathering or aging [17,45,59]. Most microplastics in the aquatic environment come from household sewage discharge, the application of sewage sludge, and the spillage of air blasting media. Secondary sources are related to littering and getting out during improper municipal solid waste collection and disposal, wind dispersal and atmospheric fallout, soil erosion, surface runoff, and stormwater runoff from urban and agricultural areas. Tire abrasion was found to be the biggest source of microplastic in many locations [31,60]. MPs reach the seas indirectly from terrestrial sources (80% of marine litter comes from terrestrial sources)—from rivers flowing in, wastewater systems, atmospheric deposition and directly from beaches and coasts, recreational and commercial fishing, marine vessels, and marine industry [17,31]. As far as nanoplastics are concerned, the sources of access to the marine ecosystems are similar (mainly form land-based input). NPs originate mostly from microplastic weathering. However, small dimensions of particles enable NPs to be transported along the food chain, in living organisms they may enter cell membranes, blood circulation, and the brain tissue [46,61].

When studying the effects of MPs and NPs on living organisms, numerous factors are taken into account, i.e., concentration, particle size, exposure time, particle condition, environmental condition, polymer type, species, developmental stage, and sex [62]. The harmful effects of microplastics and nanoplastics are mainly mechanical and toxicological in nature. They affect living organisms at different levels, reaching the cellular and molecular level [18,19,63,64]. Studies on fish have shown the effect of smaller plastic particles (i.e., size ≤ 20 μm) on behavioral and neurological functions, intestinal permeability, metabolism, and intestinal microbiome diversity [65]. The impact of NPs and MPs on human health is still insufficiently recognized [63,66]

Microplastics and nanoplastics, due to the large surface area, hydrophobicity, and high roughness surface character, have the tendency to accumulate metals, organic and biological pollution. Therefore, their harmful effects should also be considered as the carriers of chemical and biological contaminants [61,67].

Regulations on single-use plastic are on the rise globally, the restrictions on the distribution and usage of single-use plastic items are, for example: limits on the use of plastic bags through the introduction of a fee or a total sales ban, a requirement to use biodegradable straws, stirrers, and cotton buds, numerous municipalities banning the use of public funds to purchase water in plastic bottles, and introducing the requirements to provide reusable or biodegradable foodservice ware. Strict regulations apply to a growing number of countries [68,69]. The EU is the leader in solving the plastic problem; in 2019, the so-called The Single-Use Plastics Directive [69] was introduced. The directive will prohibit placing on the market 10 types of single-use plastic products (i.e., cotton buds, cutlery, plates, straws, stirrers, balloon sticks, food containers, and polystyrene cups); they will be replaced with alternative products. The regulations will come into force in 2021, so some products will disappear from the market, and for those that do not have eco-friendly substitutes, composition restrictions and appropriate recycling targets will be introduced.

The production of plastics contributes to climate change. Recent studies conducted for freshwater ecosystems indicate the interaction of climate change and eutrophication with MPs [28]. The role of microplastics as a physical, chemical, and biological stressor in a changing world that may affect ecosystems in a direct and indirect way was highlighted in the Horton and Barnes study [5]. It is crucial to thoroughly investigate the negative effects of MPs and NPs on the reduced growth of microalgae and the efficiency of photosynthesis, as microalgae provide marine ecosystem balance [70,71].

The following research needs concerning plastic waste are highlighted in the literature: bioaccumulation and biomagnification of microplastics and nanoplastics and associated chemicals in wildlife and humans [67], developing uniform methods of sample collection, separation, detection, characterization, and identification of microplastics in aqueous and solid samples; many authors stress that the lack of universal and validated methods makes the interpretation and comparing of current research results difficult [44,72,73]. Measures are also needed to reduce plastic consumption, increase recycling efficiency, drastically reduce plastic leaking to the environment, as well as the development of oceanic plastic debris clean up techniques [20,38,39,74].

## 4. Ship Industry and Marine Pollution

### 4.1. Ship Operation

**Figure 2:**
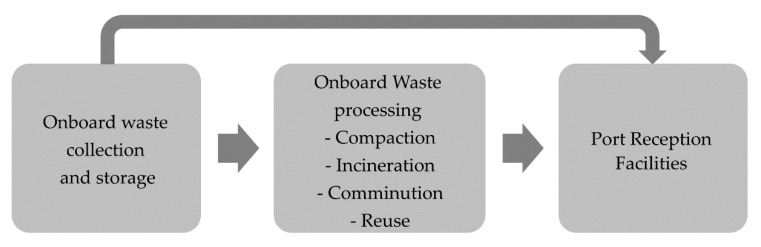
Ship-generated waste processing–waste flow diagram.

**Figure 3:**
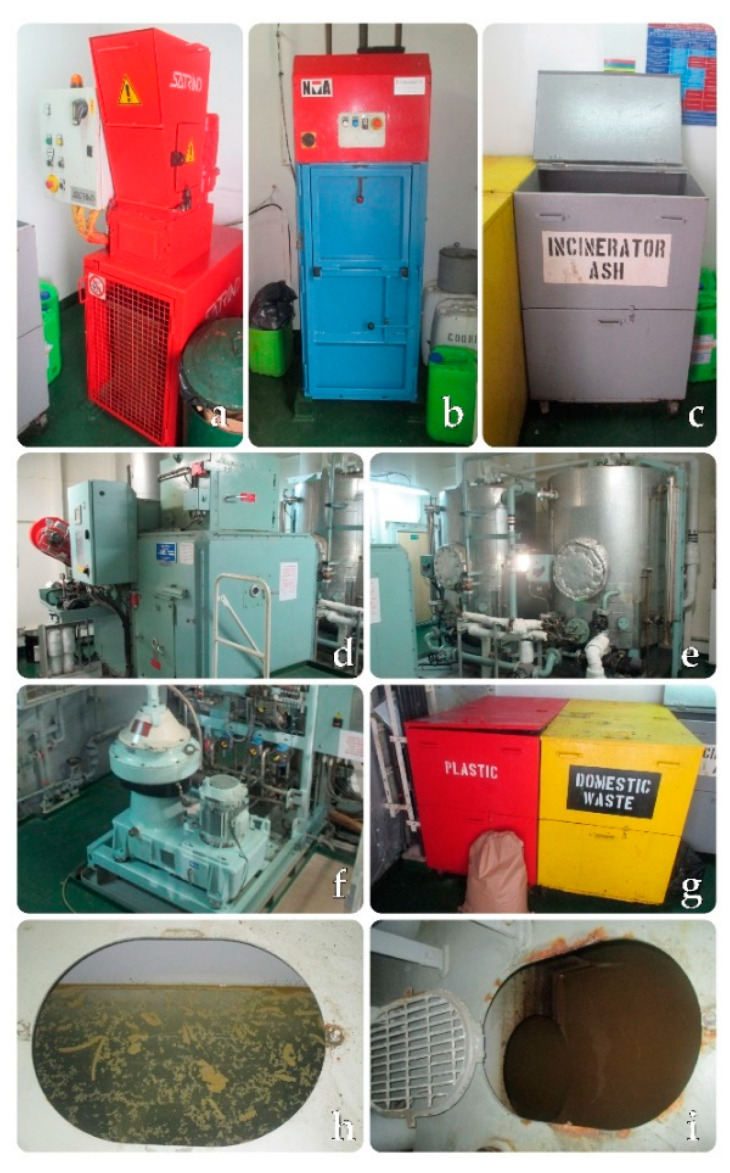
(**a**) Units involved in waste management on board ships: ship waste shredder; (**b**) ship waste compactor; (**c**,**g**) ship-generated waste collection and segregation; (**d**) ship waste incinerator; (**e**) oil derivatives processing tank; (**f**) oil separator unit; (**h**,**i**) oily bilge water and bilge well. Photo: Paweł Borowski.

Due to cost efficiency, 90–95% of food and commercial goods are transported by sea, which affects the marine environment. Maritime transport is the source of waste and pollution entering the seas and oceans in a direct way. Proper waste management on board ships is extremely important for the protection of marine ecosystems [15,75,76]. Waste generated by ships under MARPOL (The International Convention for Prevention of Marine Pollution For Ships) and the EU Directive 2000/59/E, followed by the Directive 2010/65/EU and currently in force DIRECTIVE (EU) 2019/883 OF THE EUROPEAN PARLIAMENT AND OF THE COUNCIL of 17 April 2019 must be disposed of to the Port Reception Facilities [77]. Depending on the type and size, ships may be equipped with incinerators, compactors, comminuters, or other equipment for onboard waste processing (Figure 2 and Figure 3) [78,79,80,81,82].

Waste generated on board ships which contributes to marine pollution is comprised of:

- oily residues and compounds: oily sludge, oily bilge water, waste oil, oily rags,
- plastic,
- food wastes,
- domestic wastes,
- cooking oil,
- ash from incinerators,
- operational wastes,
- cargo residues,
- ozone-depleting substances,
- other non-common waste streams [76].

MARPOL (The International Convention for Prevention of Marine Pollution For Ships) [83] is the main international convention concerning the prevention of pollution from ships caused by operational or accidental causes. It was adopted at the International Maritime Organization (IMO) in 1973.

The Convention was modified in 1978. The Protocol of 1978 was adopted in response to a number of tanker accidents in 1976–1977 and entered into force on 2 October 1983. After the revision in 1978, it is known as MARPOL 73/78. Another significant modification was made in 1997 when annex VI (Prevention of Air Pollution from Ships) was added. Amendments are made periodically through the Marine Environment Protection Committee (MEPC) of IMO [83,84,85,86].

As of now, the technical requirements of MARPOL [83] are included in six separate Annexes:

1. Annex I–Regulations for the Prevention of Pollution by Oil,
2. Annex II–Regulations for the Control of Pollution by Noxious Liquid Substances in Bulk,
3. Annex III–Prevention of Pollution by Harmful Substances Carried in Sea in Packaged Form,
4. Annex IV–Prevention of Pollution by Sewage from Ships,
5. Annex V–Prevention of Pollution by Garbage from Ships,
6. Annex VI–Prevention of Air Pollution from Ships.

According to the amendments to MARPOL Annex V which entered into force on 1 March 2018, ships need to carry:

- Placards posted on board noting the discharge requirements (applicable to ships ≥ 12 m and fixed and floating platforms),
- A Garbage Management Plan (applicable to ships ≥ 100 GT or certified to carry 15 or more persons, and fixed and floating platforms),
- A Garbage Record Book (applicable to ships ≥ 400 GT or certified to carry 15 or more persons, and fixed and floating platforms). The new format of Garbage Record Book is divided into Part I and II. Part I is applicable to all ships for recording garbage discharges of plastics, food waste, domestic wastes, cooking oil, incinerator ashes, operational waste, animal carcasses, fishing gear, and E-waste. Part II is applicable only to ships that carry solid bulk cargoes for recording discharges of cargo residues that are: cargo residues (non-HME) and cargo residues (HME).

Many authors stress the particular threat to marine environment caused by cruise ships. Cruises are nowadays one of the fastest-growing segments in the tourism sector with 27.2 million passengers in 2018 [79,87,88,89,90]. The largest vessels of this type may carry more than 6500 passengers on board and over 2000 crew, which makes them little towns on sea waters. Therefore, sustainable solutions are needed for the mitigation of the negative impact of this type of tourism on the environment. They are considered in two scenarios—sustainable technology solutions limiting the negative impact of cruise ships on the environment, or creating an alternative to cruise ships [79,89,91].

### 4.2. Ship Recycling

Ships end their economic life after 22–30 years. Due to high labor costs, care for safety conditions, and strict environmental regulations in developed countries, ship recycling takes place mainly in less developed countries located in Asia. Currently, over 80% of ships are dismantled in the countries of South Asia, in conditions that threaten the environment and the safety of workers. In addition, the development of a common policy and legislation for the recycling of end-of-life vessels is a global and controversial issue [92,93,94].

In the disposal, the owner applies procedures such as: abandonment, breaking, decommissioning, demolition, dismantling, disposal, recycling, scuttling, and scrapping [92,94]. Recycling of end-of-life vessels is performed mainly in Bangladesh, India, Pakistan, China, and Turkey. According to data from 2016, 35%, 30%, 22%, 9%, 3.7% of the gross tonnage of recycled vessels (GTRV) were placed in facilities located in these five countries, respectively, and merely 0.3% of the GTRV underwent recycling in other countries of the world [15,75,95]. When dismantling the vessel, the following on-board hazardous substances constitute a particular hazard for the environment and human health: asbestos, polychlorinated biphenyls (PCBs), glass fiber, solid foam, and waste oils [93].

The need for creating sustainable shipbreaking and ship recycling facilities (sustainable SSRI) was recognized on an international level by The Hong Kong International Convention for the Safe and Environmentally Sound Recycling of Ships adopted in 2009, which has not yet entered into force. The European Union has taken its own actions in this respect. The European Commission is working on new laws enforcing the recycling of ships in facilities that meet the requirements of the EU and have been entered in European registers. The aim is to reduce the impact of processes related to the recycling of the EU vessels on the environment and human health. The EU Ship Recycling Regulation entered into force on 30 December 2013 [92,94]. EU regulations prohibit or restrict the installation and use of hazardous materials on board ships; moreover, the European List of ship recycling facilities was created. The inventory of hazardous materials and ship recycling plan becomes an integral part of the ship recycling procedure. From 31 December 2018, large commercial EU vessels may only be recycled in safe ship recycling facilities included in the list [94,96]. Green ship recycling (GSP) means that recycling is oriented towards proper safety and environmental conditions. This requires, however, not only greater investment in the design and construction of ship recycling facilities but also in their later operation. More expensive equipment and technologies are used, and procedures related to Environmentally Sound Management are implemented. Many authors stress the need for further development of financial instruments to facilitate safe and sound ship recycling [15,94,97].

## 5. Chemical Weapon Munitions Dumped at Sea

European, Russian, Japanese, and United States coasts are the areas most affected by chemical weapons (CW) dumped into the seas (Figure 1). Although chemical weapons were used as early as the 4th century BC, the threat to the seas and oceans appeared after their mass dumping in the 20th century. After World War 2, 40,000–50,000 tonnes of chemical weapons were deposited in the Baltic Sea, including 15,000 chemical warfare agents [98,99,100]. So far, the following dumped toxic agents have been detected in marine ecosystems: Tabun, Sarin, Soman, mustard gas, nitrogen mustard, sulphur mustard, phosgene, diphosgene, chloride, chloroacetophenone, hydrogen cyanide, cyanogen chloride, arsine, Clark I, Clark II, Adamsite, Lewisite, and additives [101].

The danger posed by chemical weapons dumped in the sea is related to the progressive corrosion of barrels. The course of this process is influenced by many factors, e.g., physical and chemical properties of sea water, depth of flooding, properties of containers. It was prognosed that 2–3 cm thin steel containers can resist corrosion for up to 40–60 years, and over 70 years have passed since the end of World War 2. The second type of threat is the exploitation of marine areas, fishing (trawl nets), construction of gas pipelines, laying cables, and erecting offshore wind farms, as a result of which the containers may be damaged and toxic substances may be released. Spreading of only one-sixth of the munitions deposited in the Baltic Sea could destroy any life in this ecosystem for one hundred years [10,100,102]. The substances released are bioaccumulated in marine organisms, and the major human health risk at this time appears to arise from acute exposure to an agent by either accidental recovery or by munitions washed ashore onto beaches [98,101,102]. Although the problem of sunken chemical weapons has been known for years, there is still a knowledge deficit about the persistence, bioaccumulation, and adverse effects on humans and biota. Research is ongoing, but it is difficult to determine in detail the number and locations where CW were sunk, and many of these locations will never be recognized [10,103].

## 6. Radioactive Waste Dumping at Sea

Radioactive waste was dumped into the oceans from 1946 till 1993. The first dumping site was located in the Northeast Pacific Ocean, in the vicinity of the California coast. In 1975 the London Convention 1972 entered into force and the dumping of high-level radioactive waste at sea was banned. In 1983, a ten-year moratorium on low-level waste dumping in seas and oceans was implemented, and since 1993 there has been a total ban on such disposal of radionuclides. However, it should be added that not all countries complied with the bans and, e.g., the former Soviet Union continued dumping radioactive waste in the Arctic Seas and in the Northwest Pacific under national legislation, and this practice involved low, intermediate as well as high-level radioactive waste. The total amount of radionuclides disposed in marine ecosystems is approximately 8.5 × 104 TBq. The maximum decay corrected inventory of radioactive waste dumped at sea was reached in 1982 and was estimated at 4.5 × 104 TBq, nowadays it is less than 2.0 × 104 TBq, and it is predicted to be reduced to less than 1.0 × 104 TBq by 2050 [6,7].

A global inventory of anthropogenic radioactive material entering the marine environment is maintained by the International Atomic Energy Agency (IAEA) and includes radionuclides from two sources: (i) dumping at sea of radioactive waste, (ii) accidents and losses at sea involving actual or potential releases of radioactive material into the marine environment [7]. The report does not include radioactive substances originating from controlled discharges or accidents from land-based nuclear installations, inputs from past nuclear weapons testing and other military activities, and inputs related to natural radioactivity. According to IAEA, the sources of radioactive material entering the marine environment are as follows: waste dumping at sea including radioactive waste and packaging (nuclear reactor pressure vessels, with and without fuel; solid waste; liquid waste); moreover radioactive materials enter the sea from accidents and losses (nuclear-powered navy ships and submarines, nuclear weapons and military vessels capable of carrying such weapons, nuclear powered civilian ships, nuclear energy sources used in spacecraft, satellites and acoustic signal transmitters, radioisotope thermoelectric generators, i.e., for lighthouses power supply, sealed radiation sources). Conducted analyses showed most low- and intermediate-level radioactive waste dumped in the Atlantic and Pacific Oceans and in the Arctic Seas between 1946 and 1993 was located in Atlantic and Arctic dumping sites, the least in Pacific sites, 53.43%, 44.87%, and 1.70% of total activity, respectively. At the North Atlantic, tritium (^3^H) together with other beta and beta-gamma emitters (^90^Sr, ^134^Cs, ^l37^Cs, ^55^Fe, ^58^Co, ^60^Co, ^125^I, and ^14^C) accounted for over 98% of the total activity of the waste. The Arctic Seas were dominated by ^90^Sr and ^137^Cs, as well as ^60^Co, ^63^Ni, and ^152^Eu, representing 86% and 12% of the total inventory, respectively. Low-level radioactive waste dumped at the Northeast Atlantic sites and the high-level radioactive waste dumped by the former Soviet Union in the Arctic Seas and in the Pacific Ocean account for more than 93% of the activity of all the radioactive material dumped at sea [6,7,41].

The main sources of anthropogenic radionuclides in the Baltic Sea include discharges from nuclear facilities in the Baltic Sea basin (nuclear power plants and research reactors), discharges from nuclear reprocessing plants (Sellafield in England, and La Hague in France), the Chernobyl accident (1986), and atmospheric testing of nuclear weapons (conducted by the United States and the Soviet Union mainly in the 1950s and 1960s) [104].

Since 1963, the sinking of several nuclear submarines has been confirmed. In the Atlantic Ocean, two of them were from the USA Navy—(Thresher in 1963 and Scorpion in 1968), three others from the Soviet Union Navy (K-8 in 1970, K-219 in 1986, and K-278 Komsomolets in 1989). Two submarines belonging to the Navy of the Russian Federation sank in the Arctic Seas (K-141 Kursk in 2000, K-159 in 2003). The studies conducted so far confirmed the presence of ^60^Co in sediment samples collected in the vicinity of the Scorpion and Thresher submarines, and ^137^Cs in water and sediment samples near the wreck of the Komsomolets. Only one of the submarines, Kursk, sank at a small depth (108 m) from which it was possible to raise the wreck pieces from the sea floor in 2001, and as regards K-159, which sank at a depth of 238 m, the possibility of the salvage is being considered [7,8,105].

As mentioned above, radionuclides enter the seas and oceans as a result of accidents of nuclear power plants, the largest events of that category occurred in Chernobyl (today’s Ukraine, 1986) and in Fukushima (Japan, 2011). As a consequence of the Chernobyl nuclear power plant disaster, initial radiation exposure in contaminated areas was due to short-lived ^131^I (with a half-life of eight days), and for marine ecosystems long-lived ^137^Cs (with a half-life of 30 years) is the main hazard; moreover, in the marine ecosystems, raised concentrations of two others radionuclides present in the fallout, ^134^Cs and ^90^Sr, are observed. Presently, ^137^Cs is the main indicator of anthropogenic radioactivity in the Baltic Sea. Compared to Chernobyl, releases from Fukushima Daiichi nuclear power plant were considerably smaller (estimated at 10–15% of the Chernobyl value) [56,104,106,107]. Literature sources give divergent data on the total activity released during accidents at both power plants. An extensive analysis, comparison, and compilation of the data from different sources is found in the work by Steinhauser et al. [107]. The Chernobyl accident is responsible for 82% of ^137^Cs input to the Baltic Sea, 14% come from nuclear weapons tests, and 4% from nuclear fuel reprocessing facilities in Sellafield (England) and La Hague (France) [56]. For ^90^Sr, the proportions are reversed—81% come from the atmospheric testing of nuclear weapons and 13% from the Chernobyl release [106]. In recent years, nuclear reprocessing plants in Western Europe (Sellafield and La Hague) have become a minor source of radionuclides due to significant reductions in discharges [104].

## 7. COVID-19 Pandemic Impact on Marine Waste Generation—What We Know about Threats for Marine Ecosystems at the Beginning of 2021

### 7.1. Ship Recycling

Rahman et al. [108] studied the impact of SARS-CoV-2 pandemic on ship recycling using Weibull tonnage estimation and scenario analysis method. During the SARS-CoV-2 pandemic, the international supply chain reached a historically low level due to measures taken to minimize the spread of the virus. As a result of disruption of the global supply chain, ship recycling facilities located mainly in Asia may be affected by problems related to the lack of manpower for dismantling and disturbed transportation of ships that are to be recycled. The authors pointed out that EU ship recycling facilities can act as a buffer in the crisis. At the same time, it was emphasized that the pandemic has a varied impact on the industry and may as well be an opportunity to reframe our mode of production and consumption patterns towards more sustainable pathways.

### 7.2. Ship-Generated Waste—Fewer Cruise Ships, Less Waste

One of the first COVID-19 outbreaks happened on board the cruise ship Diamond Princess (February 2020), and in Australia, the cruise ship Ruby Princess became the largest source of coronavirus infections (March 2020). The pandemic had a very negative impact on the cruise industry, the cruises were banned for several months. It is predicted to be the biggest challenge this industry has ever faced [109]. However, the restriction of this type of tourism has had a significant influence on the reduction of the broadly understood negative impact on the environment, including the decrease of waste produced on board the ships [33,110]. The analysis shows that since March 2020 there has been an almost total collapse of the cruise market—in none of the following months was the number of 100,000 passengers exceeded, as compared to 1,600,000 passengers of this type of ships per month in the peak months of 2019 (from May to September). At the same time, a freeze in the activity of passenger ships was recorded from March to May 2020, and then the number of passengers was gradually increasing, reaching in peak months 100,000 passengers per month, similar to the amounts from 2019 [111]. Although cruise ships constitute ca. 1% of the merchant fleet, they generate over 25% of ship-sourced waste (3.5 kg/passenger/day) [112]. It can, therefore, be expected that the reduction of this type of tourism caused by COVID-19 pandemics will have a positive impact on the state of marine ecosystems.

In 2020, the guidance on the gradual and safe resumption of operations of cruise ships in the European Union in relation to the COVID-19 pandemic was created as well as disinfection of environments in healthcare and non-healthcare settings potentially contaminated with SARS-CoV-2, which points out the procedures of dealing with waste produced on board ships [113,114].

### 7.3. New Type of Marine Pollution—Coronavirus Waste

During COVID-19 pandemics, the volume of medical waste has risen up to 40%, and thus an increased generation of hazardous waste has been observed (i.e., about 600% increase in Hubei province, China) [36]. The amount of hazardous waste generated daily from each infected person is estimated to be 3.4 kg [36], in the pandemic, 129 billion face masks and 65 billion gloves are used worldwide every month [36,115], 22 kg of plastic waste is generated for every 1000 coronavirus tests (RT-PCR technique) [116]. Production of municipal waste has also increased, which has overloaded waste collection and disposal systems. The efficiency of plastic recycling has decreased substantially not only due to the inefficiency of existing installations but also in relation to the reduction of manufacturing and commercial activity—economic recession, workers of waste sector falling ill, increased recycling costs—resulting from, among other things, from the necessity to ensure workers safety and to adapt to sanitary restrictions. During the pandemic, municipalities have reduced revenues which makes the chances to invest in proper waste management slimmer. Due to the slowdown in industrial activity, the production of industrial and commercial waste has decreased [36,117].

To deal with the SARS-CoV-2, the use of Personal Protective Equipment (PPE) has increased significantly, mostly face masks, but also gloves, protective suits, boots, visors, test kits, plastic containers, bandages as well as single-use food containers and plastic bags [36]. PPE become plastic waste (macroplastics pollution in the marine environment) after usage. In 2020, a great increase in the number of news reporting the pollution of the seas and oceans by PPE waste is noted, the waste becoming a new form of pollution called coronavirus waste. PPE waste is a significant burden on the environment, particularly to the marine ecosystems [118].

Research shows that the biggest threat is posed by commonly used face masks, which may become macroplastics floating or sinking in marine waters, they are made of materials with a density both lower and higher than water, and due to their structure (non-woven fabric) they are a source of microplastics entering the environment, but also a potential vector of chemical pollutants (i.e., flame retardants) [119,120]. Production of face masks has increased more than a dozen times in recent months, in China 200 million masks were produced a day as of June, which is 20 times more than at the start of February [119,120].

COVID-19 pandemic has resulted in a significant increase in plastic waste generation worldwide (mainly due to the increased use of PPE). At the same time, the plastic recycling system has become less efficient, leading to increased plastic waste leaking to the marine environment. This may lead not only to a significant rise in the amount of waste in seas and oceans, but it may also shift the main sources of marine litter—PPE becoming the main threat [119,121]. The proper disposal of Personal Protective Equipment, as well as the development of appropriate recycling technologies for disposable (single-use) PPE used in the healthcare sector and in our homes, becomes a global challenge [35]. Rethinking and optimizing plastic waste management under the COVID-19 pandemic becomes a necessity and a technological challenge to be solved in the near future [122].

## 8. Conclusions 

The conducted analysis shows that plastic debris presently constitutes the greatest and most current threat. However, all of the abovementioned anthropogenic activities require action as a source of pollution of the seas and oceans. Reducing the negative impact of waste on the marine environment is a challenge for citizens and governments; industry including the maritime shipping industry (comprising building, registration, operation, and ship recycling); international organizations, committees, decision-makers, and regulatory bodies; worldwide NGOs and researchers. It calls for:

1. Creating and developing effective law tools related to the prevention of marine pollution (the main requirements in this field: new laws and policies, new research),
2. Promoting innovative solutions to the problem of floating marine debris and nanoplastics and microplastics. Further research on the impact of NPs and MPs on the environment and living organisms. Proper disposal, as well as the development of appropriate recycling technologies for disposable (single-use) Personal Protective Equipment (the main requirements in this field: new laws and policies, new research),
3. Safe and sustainable ship recycling. Creating financial instruments supporting Green Ship Recycling (the main requirements in this field: new laws and policies, new research),
4. Efficient management of ship-generated waste (the main requirements in this field: new laws and policies, new research),
5. Finding a solution to a problem of chemical weapons and radioactive waste dumped in the sea (the main requirements in this field: new laws and policies, new research),
6. Further studies on the influence of the COVID-19 pandemic on the marine ecosystems (the main requirements in this field: above all, new research need).

## Data Availability

Data sharing not applicable.

## References

[1] Pavlov V.K., Stanovoyà V.V. (2001). The Problem of Transfer of Radionuclide Pollution by Sea Ice. Mar. Pollut. Bull..

[2] Waller C.L., Griffiths H.J., Waluda C.M., Thorpe S.E., Loaiza I., Moreno B., Pacherres C.O., Hughes K.A. (2017). Microplastics in the Antarctic marine system: An emerging area of research. Sci. Total Environ..

[3] Lusher A.L., Tirelli V., O’Connor I., Officer R. (2015). Microplastics in Arctic polar waters: The first reported values of particles in surface and sub-surface samples. Sci. Rep..

[4] Huang D., Lin J., Du J., Yu T. (2020). The detection of Fukushima-derived radiocesium in the Bering Sea and Arctic Ocean six years after the nuclear accident. Environ. Pollut..

[5] Horton A.A., Barnes D.K.A. (2020). Microplastic pollution in a rapidly changing world: Implications for remote and vulnerable marine ecosystems. Sci. Total Environ..

[6] Sjoblom K.-L., Linsley G. (1994). Sea disposal of radioactive wastes: The London Convention 1972. IAEA Bull..

[7] IAEA (2015). Inventory of Radioactive Material Resulting from Historical Dumping, Accidents and Losses at Sea.

[8] Hu Q.-H., Weng J.-Q., Wang J.-S. (2010). Sources of anthropogenic radionuclides in the environment: A review. J. Environ. Radioact..

[9] Lebreton L., Slat B., Ferrari F., Sainte-Rose B., Aitken J., Marthouse R., Hajbane S., Cunsolo S., Schwarz A., Levivier A. (2018). Evidence that the Great Pacific Garbage Patch is rapidly accumulating plastic. Sci. Rep..

[10] Jakacki J., Andrzejewski J., Przyborska A., Muzyka M., Gordon D., Nawała J., Popiel S., Golenko M., Zhurbas V., Paka V. (2020). High resolution model for assessment of contamination by chemical warfare agents dumped in the Baltic Sea. Mar. Environ. Res..

[11] Stock F., Kochleus C., Bänsch-Baltruschat B., Brennholt N., Reifferscheid G. (2019). Sampling techniques and preparation methods for microplastic analyses in the aquatic environment—A review. TrAC Trends Anal. Chem..

[12] Martínez-Vicente V., Clark J.R., Corradi P., Aliani S., Arias M., Bochow M., Bonnery G., Cole M., Cózar A., Donnelly R. (2019). Measuring Marine Plastic Debris from Space: Initial Assessment of Observation Requirements. Remote Sens..

[13] Raha U.K., Kumar B.R., Sarkar S.K. (2021). Policy Framework for Mitigating Land-based Marine Plastic Pollution in the Gangetic Delta Region of Bay of Bengal—A review. J. Clean. Prod..

[14] Mitrano D.M., Wohlleben W. (2020). Microplastic regulation should be more precise to incentivize both innovation and environmental safety. Nat. Commun..

[15] Du Z., Zhu H., Zhou Q., Wong Y.D. (2017). Challenges and solutions for ship recycling in China. Ocean Eng..

[16] Yu Y., Zhou D., Li Z., Zhu C. (2018). Advancement and Challenges of Microplastic Pollution in the Aquatic Environment: A Review. Water Air Soil Pollut..

[17] Cole M., Lindeque P., Halsband C., Galloway T.S. (2011). Microplastics as contaminants in the marine environment: A review. Mar. Pollut. Bull..

[18] Barría C., Brandts I., Tort L., Oliveira M., Teles M. (2019). Effect of nanoplastics on fish health and performance: A review. Mar. Pollut. Bull..

[19] Wang W., Ge J., Yu X. (2020). Bioavailability and toxicity of microplastics to fish species: A review. Ecotoxicol. Environ. Saf..

[20] Bishop G., Styles D., Lens P.N.L. (2020). Recycling of European plastic is a pathway for plastic debris in the ocean. Environ. Int..

[21] Prata J.C., Silva A.L.P., da Costa J.P., Mouneyrac C., Walker T.R., Duarte A.C., Rocha-Santos T. (2019). Solutions and Integrated Strategies for the Control and Mitigation of Plastic and Microplastic Pollution. Int. J. Environ. Res. Public Health.

[22] da Silva Videla E., Vieira de Araujo F. (2021). Marine debris on the Brazilian coast: Which advances in the last decade? A literature review. Ocean Coast. Manag..

[23] Ramirez-Llodra E., Tyler P.A., Baker M.C., Bergstad O.A., Clark M.R., Escobar E., Levin L.A., Menot L., Rowden A.A., Smith C.R. (2011). Man and the Last Great Wilderness: Human Impact on the Deep Sea. PLoS ONE.

[24] Cordova M.R., Nurhati I.S. (2019). Major sources and monthly variations in the release of land-derived marine debris from the Greater Jakarta area, Indonesia. Sci. Rep..

[25] Fadeeva Z., Van Berkel R. (2021). Unlocking circular economy for prevention of marine plastic pollution: An exploration of G20 policy and initiatives. J. Environ. Manag..

[26] UNEP (2016). Marine Plastic Debris and Microplastics—Global Lessons and Research to Inspire Action and Guide Policy Change.

[27] Kunz N., Mayers K., Van Wassenhove L.N. (2018). Stakeholder Views on Extended Producer Responsibility and the Circular Economy. Calif. Manage. Rev..

[28] Zhang Y., Liang J., Zeng G., Tang W., Lu Y., Luo Y., Xing W., Tang N., Ye S., Li X. (2020). How climate change and eutrophication interact with microplastic pollution and sediment resuspension in shallow lakes: A review. Sci. Total Environ..

[29] Dabrowska J., Paweska K., Dabek P.B., Stodolak R. (2017). The implications of economic development, climate change and European water policy on surface water quality threats. Acta Sci. Pol. Circumiectus.

[30] Gall S.C., Thompson R.C. (2015). The impact of debris on marine life. Mar. Pollut. Bull..

[31] Akdogan Z., Guven B. (2019). Microplastics in the environment: A critical review of current understanding and identification of future research needs. Environ. Pollut..

[32] Szewrański S., Chruściński J., van Hoof J., Kazak J.K., Świader M., Tokarczyk-Dorociak K., Zmuda R. (2018). A location intelligence system for the assessment of pluvial flooding risk and the identification of stormwater pollutant sources from roads in suburbanised areas. Water.

[33] Braga F., Scarpa G.M., Brando V.E., Manfè G., Zaggia L. (2020). COVID-19 lockdown measures reveal human impact on water transparency in the Venice Lagoon. Sci. Total Environ..

[34] El Zowalaty M.E., Young S.G., Järhult J.D. (2020). Environmental impact of the COVID-19 pandemic—A lesson for the future. Infect. Ecol. Epidemiol..

[35] Nowakowski P., Kuśnierz S., Sosna P., Mauer J., Maj D. (2020). Disposal of Personal Protective Equipment during the COVID-19 Pandemic Is a Challenge for Waste Collection Companies and Society: A Case Study in Poland. Resources.

[36] Haque S., Uddin S., Sayem S., Mohib K.M. (2020). Coronavirus disease 2019 (COVID-19) induced waste scenario: A short overview. J. Environ. Chem. Eng..

[37] Patrício Silva A.L., Prata J.C., Walker T.R., Duarte A.C., Ouyang W., Barcelò D., Rocha-Santos T. (2021). Increased plastic pollution due to COVID-19 pandemic: Challenges and recommendations. Chem. Eng. J..

[38] Veiga J.M., Fleet D., Kinsey S., Nilsson P., Vlachogianni T., Werner S., Galgani F., Thompson R.C., Dagevos J., Gago J. (2016). Identifying Sources of Marine Litter. MSFD GES TG Marine Litter Thematic Report.

[39] World Economic Forum, Ellen MacArthur Foundation and McKinsey & Company (2016). The New Plastics Economy—Rethinking the Future of Plastics.

[40] Van Sebille E., England M.H., Froyland G. (2012). Origin, dynamics and evolution of ocean garbage patches from observed surface drifters. Environ. Res. Lett..

[41] Linsley G., Sjöblom K.-L., Cabianca T. (2005). Chapter 4 Overview of point sources of anthropogenic radionuclides in the oceans. Radioact. Environ..

[42] Wilkinson I. Chemical Weapon Munitions Dumped at Sea: An Interactive Map. http://nonproliferation.org/chemical-weapon-munitions-dumped-at-sea/.

[43] Eurostat Administrative Units/Statistical Units. https://ec.europa.eu/eurostat/web/gisco/geodata/reference-data/administrative-units-statistical-units.

[44] Fu W., Min J., Jiang W., Li Y., Zhang W. (2020). Separation, characterization and identification of microplastics and nanoplastics in the environment. Sci. Total Environ..

[45] Guo X., Wang J. (2019). The chemical behaviors of microplastics in marine environment: A review. Mar. Pollut. Bull..

[46] Dong Z., Zhu L., Zhang W., Huang R., Lv X.W., Jing X., Yang Z., Wang J., Qiu Y. (2019). Role of surface functionalities of nanoplastics on their transport in seawater-saturated sea sand. Environ. Pollut..

[47] Chen G., Feng Q., Wang J. (2020). Mini-review of microplastics in the atmosphere and their risks to humans. Sci. Total Environ..

[48] Bianco A., Passananti M. (2020). Atmospheric micro and nanoplastics: An enormous microscopic problem. Sustainability.

[49] Koelmans A.A., Mohamed Nor N.H., Hermsen E., Kooi M., Mintenig S.M., De France J. (2019). Microplastics in freshwaters and drinking water: Critical review and assessment of data quality. Water Res..

[50] Li C., Busquets R., Campos L.C. (2020). Assessment of microplastics in freshwater systems: A review. Sci. Total Environ..

[51] Zhang Z., Chen Y. (2020). Effects of microplastics on wastewater and sewage sludge treatment and their removal: A review. Chem. Eng. J..

[52] Ruimin Q., Jones D.L., Zhen L., Qin L., Changrong Y. (2020). Behavior of microplastics and plastic film residues in the soil environment: A critical review. Sci. Total Environ..

[53] Pathan S.I., Arfaioli P., Bardelli T., Ceccherini M.T., Nannipieri P., Pietramellara G. (2020). Soil Pollution from Micro- and Nanoplastic Debris: A Hidden and Unknown Biohazard. Sustainability.

[54] Khalid N., Aqeel M., Noman A. (2020). Microplastics could be a threat to plants in terrestrial systems directly or indirectly. Environ. Pollut..

[55] Du C., Liang H., Li Z., Gong J. (2020). Pollution Characteristics of Microplastics in Soils in Southeastern Suburbs of Baoding City, China. Int. J. Environ. Res. Public Health.

[56] Saremi S., Isaksson M., Harding K.C. (2018). Bio accumulation of radioactive caesium in marine mammals in the Baltic Sea—Reconstruction of a historical time series. Sci. Total Environ..

[57] Devault D.A., Beilvert B., Winterton P. (2017). Ship breaking or scuttling? A review of environmental, economic and forensic issues for decision support. Environ. Sci. Pollut. Res..

[58] Ajith N., Arumugam S., Parthasarathy S., Manupoori S., Janakiraman S. (2020). Global distribution of microplastics and its impact on marine environment—A review. Environ. Sci. Pollut. Res..

[59] Qin F., Du J., Gao J., Liu G., Song Y., Yang A., Wang H., Ding Y., Wang Q. (2020). Bibliometric Profile of Global Microplastics Research from 2004 to 2019. Int. J. Environ. Res. Public Health.

[60] Sommer F., Dietze V., Baum A., Sauer J., Gilge S., Maschowski C., Gieré R. (2018). Tire abrasion as a major source of microplastics in the environment. Aerosol Air Qual. Res..

[61] Shen M., Zhang Y., Zhu Y., Song B., Zeng G., Hu D., Wen X., Ren X. (2019). Recent advances in toxicological research of nanoplastics in the environment: A review. Environ. Pollut..

[62] Kögel T., Bjorøy Ø., Toto B., Bienfait A.M., Sanden M. (2020). Micro- and nanoplastic toxicity on aquatic life: Determining factors. Sci. Total Environ..

[63] Yong C.Q.Y., Valiyaveetill S., Tang B.L. (2020). Toxicity of Microplastics and Nanoplastics in Mammalian Systems. Int. J. Environ. Res. Public Health.

[64] Liu Z., Cai M., Wu D., Yu P., Jiao Y., Jiang Q., Zhao Y. (2020). Effects of nanoplastics at predicted environmental concentration on Daphnia pulex after exposure through multiple generations. Environ. Pollut..

[65] Jacob H., Besson M., Swarzenski P.W., Lecchini D., Metian M. (2020). Effects of Virgin Micro- and Nanoplastics on Fish: Trends, Meta-Analysis, and Perspectives. Environ. Sci. Technol..

[66] Banerjee A., Shelver W.L. (2021). Micro- and nanoplastic induced cellular toxicity in mammals: A review. Sci. Total Environ..

[67] Peng J., Wang J., Cai L. (2017). Current understanding of microplastics in the environment: Occurrence, fate, risks, and what we should do. Integr. Environ. Assess. Manag..

[68] Chen Y., Awasthi A.K., Wei F., Tan Q., Li J. (2021). Single-use plastics: Production, usage, disposal, and adverse impacts. Sci. Total Environ..

[69] Official Journal of the European Union (2019). Directive (EU) 2019/904 of the European Parliament and of the Council of 5 June 2019 on the Reduction of the Impact of Certain Plastic Products on the Environment.

[70] Zhang C., Chen X., Wang J., Tan L. (2017). Toxic effects of microplastic on marine microalgae Skeletonema costatum: Interactions between microplastic and algae. Environ. Pollut..

[71] Gangadoo S., Owen S., Rajapaksha P., Plaisted K., Cheeseman S., Haddara H., Truong V.K., Ngo S.T., Vu V.V., Cozzolino D. (2020). Nano-plastics and their analytical characterisation and fate in the marine environment: From source to sea. Sci. Total Environ..

[72] Thomas D., Schütze B., Heinze W.M., Steinmetz Z. (2020). Sample Preparation Techniques for the Analysis of Microplastics in Soil—A Review. Sustainability.

[73] Prata J.C., da Costa J.P., Duarte A.C., Rocha-Santos T. (2019). Methods for sampling and detection of microplastics in water and sediment: A critical review. TrAC Trends Anal. Chem..

[74] Shaw H.J., Chen W.L., Li Y.H. (2019). A CFD study on the performance of a passive ocean plastic collector under rough sea conditions. Ocean Eng..

[75] Eronat A.H., Bengil F., Neşer G. (2019). Shipping and ship recycling related oil pollution detection in Çandarlı Bay (Turkey) using satellite monitoring. Ocean Eng..

[76] CE Delft (2017). The Management of Ship-Generated Waste On-Board Ships EMSA/OP/02/2016.

[77] Official Journal of the European Union (2019). Directive (EU) 2019/883 of the European Parliament and of the Council of 17 April 2019 on Port Reception Facilities for the Delivery of Waste from Ships, Amending Directive 2010/65/EU and Repealing Directive 2000/59/EC.

[78] Vaneeckhaute C., Fazli A. (2020). Management of ship-generated food waste and sewage on the Baltic Sea: A review. Waste Manag..

[79] Sanches V.M.L., Aguiar M.R.d.C.M., de Freitas M.A.V., Pacheco E.B.A.V. (2020). Management of cruise ship-generated solid waste: A review. Mar. Pollut. Bull..

[80] Vaneeckhaute C., Darveau O. (2020). Current state and potential valorisation of ship-generated organic waste in Quebec, Canada. Waste Manag..

[81] Carpenter A., Macgill S.M. (2005). The EU Directive on port reception facilities for ship-generated waste and cargo residues: The results of a second survey on the provision and uptake of facilities in North Sea ports. Mar. Pollut. Bull..

[82] MEPC (2012). Resolution MEPC.219(63) Adopted on 2 March 2012.

[83] IMO (1973). International Convention for the Prevention of Pollution from Ships.

[84] IMO (2019). I666E IMO 2020: Consistent Implementation of MARPOL Annex VI.

[85] IMO (2017). IE520E MARPOL Consolidated Edition.

[86] Uche-Soria M., Rodríguez-Monroy C. (2019). Solutions to Marine Pollution in Canary Islands’ ports: Alternatives and Optimization of Energy Management. Resources.

[87] Armellini A., Daniotti S., Pinamonti P., Reini M. (2019). Reducing the environmental impact of large cruise ships by the adoption of complex cogenerative/trigenerative energy systems. Energy Convers. Manag..

[88] Toneatti L., Deluca C., Fraleoni-Morgera A., Pozzetto D. (2020). Rationalization and optimization of waste management and treatment in modern cruise ships. Waste Manag..

[89] Carić H. (2016). Challenges and prospects of valuation—Cruise ship pollution case. J. Clean. Prod..

[90] Slišković M., Ukić Boljat H., Jelaska I., Jelić Mrčelić G. (2018). Review of Generated Waste from Cruisers: Dubrovnik, Split, and Zadar Port Case Studies. Resources.

[91] Pesce M., Terzi S., Al-Jawasreh R.I.M., Bommarito C., Calgaro L., Fogarin S., Russo E., Marcomini A., Linkov I. (2018). Selecting sustainable alternatives for cruise ships in Venice using multi-criteria decision analysis. Sci. Total Environ..

[92] Ignacio Alcaide J., Rodríguez-Díaz E., Piniella F. (2017). European policies on ship recycling: A stakeholder survey. Mar. Policy.

[93] Du Z., Zhang S., Zhou Q., Yuen K.F., Wong Y.D. (2018). Hazardous materials analysis and disposal procedures during ship recycling. Resour. Conserv. Recycl..

[94] Schøyen H., Burki U., Kurian S. (2017). Ship-owners’ stance to environmental and safety conditions in ship recycling. A case study among Norwegian shipping managers. Case Stud. Transp. Policy.

[95] (2017). NGO Shipbreaking Platform. Substandard Shipbreaking: A Global Challenge.

[96] Devaux C., Nicolaï J.P. (2020). Designing an EU Ship Recycling Licence: A Roadmap. Mar. Policy.

[97] Rahman A., Karim M.M. (2015). Green Shipbuilding and Recycling: Issues and Challenges. Int. J. Environ. Sci. Dev..

[98] Greenberg M.I., Sexton K.J., Vearrier D. (2016). Sea-dumped chemical weapons: Environmental risk, occupational hazard. Clin. Toxicol..

[99] Vanninen P., Östin A., Bełdowski J., Pedersen E.A., Söderström M., Szubska M., Grabowski M., Siedlewicz G., Czub M., Popiel S. (2020). Exposure status of sea-dumped chemical warfare agents in the Baltic Sea. Mar. Environ. Res..

[100] HELCOM (2013). Chemical Munitions Dumped in the Baltic Sea. Report of the Ad Hoc Expert Group to Update and Review the Existing Information on Dumped Chemical Munitions in the Baltic Sea (HELCOM MUNI), Baltic Sea Environment Proceeding (BSEP) No. 142.

[101] Szarejko A., Namieśnik J. (2009). The Baltic Sea as a dumping site of chemical munitions and chemical warfare agents. Chem. Ecol..

[102] Glasby G.P. (1997). Disposal of chemical weapons in the Baltic Sea. Sci. Total Environ..

[103] Tornero V., Hanke G. (2016). Chemical contaminants entering the marine environment from sea-based sources: A review with a focus on European seas. Mar. Pollut. Bull..

[104] HELCOM (2009). Radioactivity in the Baltic Sea, 1999–2006.

[105] Ølgaard P.L. (1996). Nordisk Kernesikkerhedsforskning. Accidents in Nuclear Ships.

[106] Zalewska T., Suplińska M. (2013). Anthropogenic radionuclides 137Cs and 90Sr in the southern Baltic Sea ecosystem. Oceanologia.

[107] Steinhauser G., Brandl A., Johnson T.E. (2014). Comparison of the Chernobyl and Fukushima nuclear accidents: A review of the environmental impacts. Sci. Total Environ..

[108] Rahman S.M.M., Kim J., Laratte B. (2021). Disruption in Circularity? Impact analysis of COVID-19 on ship recycling using Weibull tonnage estimation and scenario analysis method. Resour. Conserv. Recycl..

[109] Ito H., Hanaoka S., Kawasaki T. (2020). The cruise industry and the COVID-19 outbreak. Transp. Res. Interdiscip. Perspect..

[110] STAND (2020). Earth COVID Pandemic Results in a Cleaner Coast. An Investigation into Unregulated Cruise Ship Pollution in Canada’s West Coast Waters.

[111] EMSA (2020). COVID-19—Impact on Shipping. November 2020.

[112] Butt N. (2007). The impact of cruise ship generated waste on home ports and ports of call: A study of Southampton. Mar. Policy.

[113] EMSA (2020). COVID-19: EU Guidance for Cruise Ship Operations. Guidance on the Gradual and Safe Resumption of Operations of Cruise Ships in the European Union in Relation to the COVID-19 Pandemic.

[114] ECDC (2020). Disinfection of Environments in Healthcare and Non-Healthcare Settings Potentially Contaminated with SARS-CoV-2.

[115] Prata J.C., Silva A.L.P., Walker T.R., Duarte A.C., Rocha-Santos T. (2020). COVID-19 Pandemic Repercussions on the Use and Management of Plastics. Environ. Sci. Technol..

[116] Parashar N., Hait S. (2021). Plastics in the time of COVID-19 pandemic: Protector or polluter?. Sci. Total Environ..

[117] Rajesh S., James D.M. (2020). Elcin Akcura & Lamin Njie. COVID-19’s Impact on the Waste Sector.

[118] Cordova M.R., Nurhati I.S., Riani E., Nurhasanah, Iswari M.Y. (2021). Unprecedented plastic-made personal protective equipment (PPE) debris in river outlets into Jakarta Bay during COVID-19 pandemic. Chemosphere.

[119] De-la-Torre G.E., Aragaw T.A. (2020). What we need to know about PPE associated with the COVID-19 pandemic in the marine environment. Mar. Pollut. Bull..

[120] Aragaw T.A. (2020). Surgical face masks as a potential source for microplastic pollution in the COVID-19 scenario. Mar. Pollut. Bull..

[121] Canning-Clode J., Sepúlveda P., Almeida S., Monteiro J. (2020). Will COVID-19 Containment and Treatment Measures Drive Shifts in Marine Litter Pollution?. Front. Mar. Sci..

[122] Patrício Silva A.L., Prata J.C., Walker T.R., Campos D., Duarte A.C., Soares A.M.V.M., Barcelò D., Rocha-Santos T. (2020). Rethinking and optimising plastic waste management under COVID-19 pandemic: Policy solutions based on redesign and reduction of single-use plastics and personal protective equipment. Sci. Total Environ..

